# Integration of suboptimal health status and endothelial dysfunction as a new aspect for risk evaluation of cardiovascular disease

**DOI:** 10.1186/s13167-016-0068-0

**Published:** 2016-09-12

**Authors:** Vitalii Kupaev, Oleg Borisov, Ekaterina Marutina, Yu-Xiang Yan, Wei Wang

**Affiliations:** 1 Samara State Medical University, Samara, Russian Federation; 2 Department of Epidemiology and Biostatistics, School of Public Health, Capital Medical University, Beijing, 100069 China; 3 Postgraduate Medicine, School of Medical Sciences and Health, Edith Cowan University, Perth, Australia

**Keywords:** Suboptimal health status, Endothelial dysfunction, Risk factors, Cardiovascular disease

## Abstract

**Background:**

Suboptimal health status (SHS) is recognized as a subclinical, reversible stage of chronic disease. Association has been confirmed between SHS and cardiovascular risk factors, indicating that SHS may contribute to the development of cardiovascular disease. This study explored integrated risk assessment of cardiovascular disease by combining SHS questionnaire-25 (SHSQ-25) and indicators of endothelial dysfunction.

**Methods:**

A community-based cross-sectional study was conducted in a sample of 459 residents of Samara, Russia, who had no history of clinical diagnosed disease and did not receive any treatment in the last 2 weeks. The SHS score was derived from the data collected in the SHSQ-25. Blood pressure, body mass index, and glucose and lipid levels (total cholesterol, low density lipoprotein, cholesterol and triglycerides) were measured by physical examination and laboratory performance. The relationship between SHS and endothelial dysfunction was examined using Pearson’s correlation linear regression analysis. Cluster analysis was performed to identify systemic patterns arising from exposure to a variety of risk factors.

**Results:**

Significant correlations were observed between index of endothelial function and the overall performance of SHS (*r* = −0.31, *p* < 0.05), and individual scales of the questionnaire SHSQ-25: fatigue (*r* = −0.36, *p* < 0.05), mental (*r* = −0.29, *p* < 0.05), and the cardiovascular system (*r* = −0.36). Based on cluster analysis, all subjects were grouped into five clusters: (1) optimal health status, (2) SHS at low risk of disease states, (3) SHS with a high risk of non-cardiac pathologies profile, (4) SHS of low risk of cardiovascular disease, and (5) SHS with high risk of cardiovascular disease.

**Conclusions:**

SHS is associated with endothelial dysfunction. Integration of suboptimal health status and endothelial dysfunction provides a novel tool to allow people to get a more holistic picture of both subjective and objective health measures, and also can be applied to routine screening for risks of cardiovascular diseases.

## Background

In the past 20 years, economic growth and associated sociodemographic changes in the Russian Federation have increased the prevalence of the major risk factors for chronic disease, which leads to an increased morbidity and mortality, especially for cardiovascular disease [1, 2]. Preclinical status of diseases and its early detection have become major issues in the promotion of the basic health service in the reform of health care. It is necessary that an economical and valid instrument is developed as screening technique. Among the new methods of questionnaire screening, estimate of suboptimal health status (SHS) deserves special attention, which was first developed, tested, and validated in China [3] (Appendix). This technique opens up new possibilities in the study of risk factors and early symptoms of many diseases, cardiovascular diseases, in particular [4].

SHS is characterized by ambiguous health complaints, general weakness and lack of vitality, which is regarded as subclinical, reversible stage of the disease [3–5]. The studies show that SHS may contribute to the progression or development of chronic disease [5]. Linear two-level models have revealed that SHS is associated with cardiovascular risk factors including increased blood pressure and total cholesterol and decreased high density lipoprotein, cholesterol in either men or women [4].

As the unifying concept, endothelium is a target for prevention and treatment of pathological processes leading or implementing cardiovascular disease. Vessel stiffness is an integral index, determined not only to the structural elements of the vascular wall and blood pressure but also to the regulatory mechanisms, among which endothelial dysfunction plays a key role [6–8].

In this study, we explored integrated risk assessment of cardiovascular disease by combining SHS questionnaire-25 (SHSQ-25) and indicators of endothelial dysfunction.

## Methods

### Study participants

A cross-sectional study was conducted among 459 residents of the city of Samara, Russia based on random cluster sampling with district as the basic sampling unit. Participants had no history of clinical diagnosed disease and did not receive any treatment in the last 2 weeks. The investigation was carried out during the professional examinations in the clinic of Samara State Medical University and the health centers in its tertiary teaching hospitals in Samara. To assure comparability of the findings, all participants were examined by the physicians who were specially trained for the study. Both the university and hospitals’ research ethical committees approved the study.

### Data collection

A self-reported questionnaire SHSQ-25 was used to assess SHS [3]. The questionnaire included 25 items and encompassed five subscales: fatigue (9 items: 1–6, 8–10), cardiovascular system (3 items: 11–13), digestive tract (3 items: 14–16), immune system (3 items: 7, 17, 25), and mental status (7 items: 18–24). Each subject was asked to rate a specific statement on a 5-point Likert-type scale based on how often they suffered various specific complaints in the preceding 3 months: (1) never or almost never, (2) occasionally, (3) often, (4) very often, and (5) always. The raw scores of 1 to 5 on the questionnaire were recoded as 0 to 4. SHS scores were calculated for each respondent by summing the ratings for the 25 items. A high score represents a high level of SHS (poor health). The total score of more than 35 points indicates state of suboptimal status, which would require a more in-depth survey of all five scales [9, 10].

Body weight and height were measured twice during the interview. The averages were used in the analysis. Weight was measured in light indoor clothing without shoes on electronic scales placed on a firm, level surface to the nearest 0.1 kg. Height was measured without shoes with a wall-mounted stadiometer to the nearest 0.1 cm. Body mass index (BMI) was calculated as body weight (in kilograms) divided by height (in meters) squared. Using a standard mercury sphygmomanometer, blood pressure was measured three times consecutively after 5-min rest, with each participant seated. The average of the second and third measurements was used to estimate the systolic blood pressures (SBP) and diastolic blood pressures (DBP). Smoking index was defined as the ratio of cigarettes smoked per day by the general experience of smoking in years.

Overnight fasting blood specimens were obtained for the measurement of plasma glucose and serum lipids. Plasma glucose was measured using a glucose-oxidase enzymatic method (Bechman Coulter Uni Cel DcX 600i). Concentrations of total cholesterol (TCH), high density lipoprotein (HDL) cholesterol, low-density lipoprotein (LDL) cholesterol, and triglycerides (TG) were assessed enzymatically with commercially available reagents («LDL-C Select FS DiaSys» DiaSys Diagnostic Systems GmbH & Co.KG, Germany).

Indicators of arterial stiffness and endothelial dysfunction were determined using the standard brachial plethysmography technique “Eldar” [11] (New Devices, Samara, Russia) in which forearm blood flow responses to brachial artery infusions of acetylcholine (ACh; 7.5–15.0 μg/min) and sodium nitroprusside (NP; 3–10 μg/min) were obtained and could be expressed in absolute terms or as the ACh/NP ratio. Results were referenced by comparison with flow in the other arm. The index of reflection (IR): the ratio of the amplitude of the reflected pulse wave A2 to A1 forward wave amplitude, expressed as a percentage; stiffness index (IS): the ratio of the length of the body of the subject (in meters) at the time of the pulse wave reflection (seconds) and the index of endothelial function (IEF): the value of the index reflects changes in the course of reactive hyperemia in the third minute post occlusion blood flow (IR-3 min) compared to baseline prior to the test.

### Statistical analyses

Data were reported as mean ± SD for continuous variables and frequencies for categorical variables. The subjects were classified into two groups: group 1: healthy (*n* = 295), group 2 (*n* = 164): persons with risk classical factors for cardiovascular disease, i.e., blood pressure (>140/90 mmHg), glucose (>6.1 mmol/l), lipid levels (TCH >5 mmol/l), and BMI (>24.5 kg/m^2^). Student’s *t* test was used to compare the difference of the scores on SHSQ-25 between the two groups. The relationship between SHS and endothelial dysfunction was examined using Pearson’s correlation analysis and multiple linear regression analysis (MLR) with SHSQ scores as the dependent variable and endothelial dysfunction as the independent variable. Cluster analysis was performed to identify systemic patterns arising from exposure to a variety of risk factors. The subjects were identified as separate local subsets (clusters) according to grouping attributes with k-means clustering. A *p* value of less than 0.05 was considered to be of statistical significance. All *p* values reported were two-tailed. All statistical analyses were performed using software package “Statistica 8.0” (Statsoft, Tulsa, USA).

## Results

All 459 participants completed the questionnaire and laboratory and instrumental measurements. Their mean age was 34.01 years (SD 14.10) and 58.6 % were women. The participants among group with cardiovascular risk showed higher proportions of male (51.2 vs. 35.9 %, *p* < 0.001), older age (74.4 vs. 13.2 %, *p* < 0.001), and labor workers (51.83 vs. 8.5 %, *p* < 0.001) than those among the healthy group (Table 1).

**Table 1:** Characteristics of the studied groups

Variables	Healthy group (*n* = 295)	Group with cardiovascular risk (*n* = 164)	*χ* ^2^	*p*
Gender				
Male	106 (35.9 %)	84 (51.2 %)	10.15	<0.01
Female	189 (64.1 %)	80 (48.8 %)		
Age				
18–40 years	256 (86.8 %)	42 (25.6 %)	173.19	<0.01
41–60 years	39 (13.2 %)	122 (74.4 %)		
Occupation				
Office workers	267 (90.5 %)	79 (48.17 %)	101.81	<0.01
Labor workers	28 (8.5 %)	85 (51.83 %)		
Suboptimal health status	23 (7.8 %)	14 (8.8 %)	0.029	>0.05

There were no significant differences in the proportion of individuals with suboptimal status, between the healthy group and the group of cardiovascular risk (7.8 vs. 8.8 %, *p* > 0.05). The total score of SHSQ-25 were 14.92 ± 9.14 among the healthy group and 16.42 ± 9.07 among the group of cardiovascular risk, which did not show significant difference among the two groups (*p* = 0.09). However, SHSQ-25 questionnaire showed differences between the two groups on the three sub-scales of suboptimal status, i.e., (1) fatigue, (2) cardiovascular system, and (3) immune system (*p* < 0.05). The largest discrepancy focused on the sub-scales of the cardiovascular system and fatigue. The average scale score of the cardiovascular system among subjects with cardiovascular risk was 1.95 ± 1.81, which was significantly higher than that of the control group (*p* < 0.001), whose score was 0.90 ± 1.2.

The group with risk factors for cardiovascular disease had significantly higher levels of SBP, DBP, BMI, smoking index, TCH, TG, and LDL cholesterol (Table 2).

**Table 2:** Comparison of the cardiovascular risk factors between healthy group and group with cardiovascular risk

Variables	Healthy group	Group with cardiovascular risk	*t*	*p*
	Mean + Std.	Mean + Std.		
Smoking index	1.66 ± 10.2	7.9 ± 15.5	4.801	<0.001
BMI (kg/m^2^)	22.1 ± 4.2	28.55 ± 5.0	14.12	<0.001
SBP (mmHg)	111.73 ± 12.7	129.5 ± 11.9	13.611	<0.001
DBP (mmHg)	73.86 ± 8.2	80.86 ± 8.3	8.803	<0.001
GLU (mmol/L)	4.03 ± 0.86	4.67 ± 0.96	6.403	<0.001
TCH (mmol/L)	4.28 ± 0.8	5.6 ± 0.96	6.441	<0.001
TG (mmol/L)	1.11 ± 0.57	1.56 ± 0.87	2.801	0.010
HDL (mmol/L)	1.36 ± 0.36	1.32 ± 0.31	3.230	<0.001
LDL (mmol/L)	2.85 ± 0.17	3.01 ± 0.37	2.562	0.006

*BMI* body mass index, *SBP* systolic blood pressure, *DBP* diastolic blood pressure, *GLU* plasma glucose, *TCH* total cholesterol, *TG* triglyceride, *LDLC* low-density lipoprotein cholesterol

## Discussion

Changes in lipid affect the condition of vessel wall—the endothelium. Over the last decade, there is accumulated evidence of the importance of determining the stiffness of the arterial wall as an indicator on vascular remodeling. With photopletismography arterial stiffness and endothelial dysfunction can be accessed. The higher the absolute value of stiffness index, the lower the expression of index of endothelial function (IEF) and a healthy vascular wall. Our study showed that in the group of participants with risk factors value was 7.5 ± 7.6 %, which was significantly lower (*p* < 0.01) than those in healthy group, whose proportion was 18.4 ± 7.7 %. The index of endothelial function was found significantly correlated with the overall performance of suboptimal health status (*r* = −0.31, *p* < 0.05), as well as with individual sub-scales of the questionnaire SHSQ-25: fatigue (*r* = −0.36, *p* < 0.05), mental (*r* = −0.29, *p* < 0.05), and the cardiovascular system (*r* = −0.36, *p* < 0.05). Linear regression also showed association between SHS and IEF (Table 3).

**Table 3:** The results of the regression analysis (dependent variable SHSQ score)

Variables	Unstandardized coefficients		Standardized coefficients	*t*	*p*
	Beta	Std. error	Beta		
Age	−0.132	0.053	−0.209	−2.491	0.013
Smoking index	0.034	0.085	0.026	0.398	0.691
BMI	0.139	0.145	0.079	0.958	0.339
SBP	0.069	0.054	0.102	1.288	0.199
DBP	−0.040	0.049	0.052	−0.815	0.416
IEF	−0.248	0.068	−0.284	−3.679	0.000

*BMI* body mass index, *SBP* systolic blood pressure, *DBP* diastolic blood pressure, *IEF* index of endothelial function

Given the obvious correlations between indicators of endothelial dysfunction and the values of the scales of the questionnaire SHSQ-25, we explored the integral relationship between the values of SHS indicators of endothelial dysfunction and risk factors for cardiovascular disease. In our study, a newly created instrument, SHSQ-25, was used for measurement of SHS. The SHSQ-25 is a self-rated questionnaire of perceived health complaints which is a brief and valid instrument for the assessment of SHS [3]. To do this, we used multivariate statistical analysis on the following parameters: the values of profiles SHS-25 subscale (“fatigue,” “mental status,” “cardiovascular system,” “digestive system,” “immune system,” and “the total amount of SHS-25”), the index of the smoker, BMI, SBP, DBP and endothelial function parameters, vascular stiffness index, the index of reflection pulse wave, blood glucose, and TCH.

**Fig. 1:**
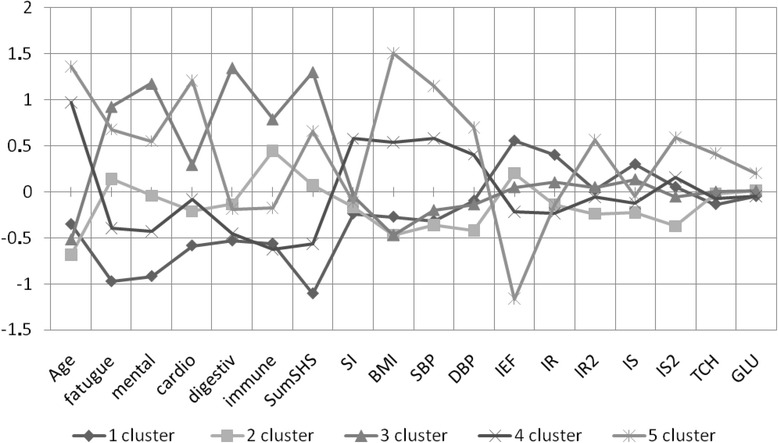
Cluster analysis of integration of suboptimal health status, cardiovascular risk, and endothelial dysfunction. The figure shows a graph obtained during cluster analysis, allowing for classification into clusters after pre-processing the received data in accordance with the average values in the whole population. *SumSHS: the total amount of SHS score: 25. *SI* smoker index, *BMI* body mass index, *SBP* systolic blood pressures, *DBP* diastolic blood pressures, *IEF* index of endothelial function, *IR* index of reflection before the sample, *IR2* index of reflection after the sample, *IS* stiffness index before the sample, *IS2* stiffness index after the sample, *TCH* total cholesterol, *GLU* glucose

Based on the cluster analysis on risk factors of cardiovascular system and indicators of SHS, all the subjects were classified into five clusters (Fig. 1). The first cluster includes 99 young persons, with a low value of the total index SHSQ-25, normal weight, blood pressure, lack of endothelial dysfunction, reduced levels of glucose and cholesterol. These persons were estimated as the persons with the optimal health status. The second cluster contains 121 cases. This cluster was characterized by the young age of the participants, the mean value of the total index SHSQ-25, with deviations in the mental sphere, and the immune system, normal weight, blood pressure, lack of endothelial dysfunction, reduced levels of glucose, cholesterol. This cluster was described as a cluster of SHS at low risk of disease states. The third cluster (*n* = 91 cases) is different from the other two by high values of the cumulative index SHSQ-25, especially on the scale of the mental sphere, digestive tract, and immune system. This cluster was described by us as a cluster of SHS with a high risk of non-cardiac pathologies profile. Faces of the fourth cluster (*n* = 94 cases) were aged over 35 years, with the average values of the total index SHSQ-25, but with the presence of at least 1–2 risk factors for cardiovascular disease. This is mainly overweight or long smoking history. We designated it as a cardiovascular phenotype of SHS of low risk of cardiovascular disease. And finally, the fifth cluster (*n* = 54 cases) differs in significant variations in the total index SHSQ-25, the scale of cardiovascular disease, and the presence of risk factors for cardiovascular disease and endothelial dysfunction. These patients were referred to our cardiovascular phenotype of SHS with high risk of cardiovascular disease.

Cluster analysis showed a correlation between SHS score (including total index SHSQ-25 and sub-scales of the SHSQ-25), risk factors for cardiovascular system, and indicators of endothelial dysfunction (*p* < 0.001). Among the risk factors for cardiovascular diseases, the greatest distance between the clusters 1, 2, 3 on one side and 4, 5 clusters on the other side were observed according to age, body mass index, and blood pressure, indicating that the association between stiffness of vascular wall with a number of traditional determinants of cardiovascular diseases at suboptimal health stage.

The present study combined evaluation of suboptimal health status with the analysis of the state of endothelial dysfunction, which allows us to identify the risk of developing cardiovascular disease, which enables people early intervention in terms of predictive, preventive, and personalized medicine [12, 13]. However, there are limitations of the present study: information of physical activity habits, dietary profile, meals frequency, and sleep habits have not been assessed. All these parameters are relevant with health status measurements [14, 15].

## Conclusions

1. Suboptimal health status is associated with risk factors of cardiovascular disease. Effective intervention on SHS may be a cost-effective way for preventing cardiovascular disease.
2. The evaluation of suboptimal health status, combined with the analysis of the state of endothelial dysfunction allows identifying the risk of developing cardiovascular disease, which provides people holistic picture of both subjective and objective health measures from the perspective of PPPM.

## Appendix A

### Sub-health status questionnaire (SHSQ-25)

The following questions ask some events about your health during the last 3 months.

Answer every question by making the appropriate box with an “x”. You may choose from one of the following answers:

**Table:**

1	2	3	4	5	
never or almost never	now and then	often	very often	always	
How often is it, that you (your)	1	2	3	4	5
1. were exhausted without physical actives significantly increasing	□	□	□	□	□
2. fatigue could not be substantially alleviated by rest	□	□	□	□	□
3. were languid when working	□	□	□	□	□
4. suffered from headaches	□	□	□	□	□
5. suffered from dizziness	□	□	□	□	□
6. eyes were aching and tired	□	□	□	□	□
7. suffered from sore throat	□	□	□	□	□
8. muscles or joints felt stiff	□	□	□	□	□
9. have pains in shoulder / neck / waist	□	□	□	□	□
10. have heavy feeling in legs when walking	□	□	□	□	□
11. got out of breath while sitting still	□	□	□	□	□
12. suffered from sore throat	□	□	□	□	□
13. were bothered by heart palpitation	□	□	□	□	□
14. got poor appetite	□	□	□	□	□
15. suffered from an upset stomach	□	□	□	□	□
16. suffered from indigestion	□	□	□	□	□
17. got tender fever or cold in-tolerance	□	□	□	□	□
18. had difficulty in falling asleep	□	□	□	□	□
19. had trouble with waking up during night	□	□	□	□	□
20. had trouble with impairment in short memory	□	□	□	□	□
21. could not respond quickly	□	□	□	□	□
22. had difficulty in concentration	□	□	□	□	□
23. were distracted for no reason	□	□	□	□	□
24. were keyed up or jittery	□	□	□	□	□
25. were caught with colds in the past 1 year	□	□	□	□	□
